# Toxicological properties of emission particles from heavy duty engines powered by conventional and bio-based diesel fuels and compressed natural gas

**DOI:** 10.1186/1743-8977-9-37

**Published:** 2012-09-29

**Authors:** Pasi I Jalava, Päivi Aakko-Saksa, Timo Murtonen, Mikko S Happo, Ari Markkanen, Pasi Yli-Pirilä, Pasi Hakulinen, Risto Hillamo, Jorma Mäki-Paakkanen, Raimo O Salonen, Jorma Jokiniemi, Maija-Riitta Hirvonen

**Affiliations:** 1 University of Eastern Finland, Department of Environmental Science, Kuopio, Finland; 2 VTT Technical Research Centre of Finland, Espoo, Finland; 3 National Institute for Health and Welfare, Department of Environmental Health, Kuopio, Finland; 4 Finnish Meteorological Institute, Air Quality Research, Helsinki, Finland

**Keywords:** Biodiesel, Hydrotreated vegetable oil, Particulate matter, Emissions, In vitro toxicology, Compressed natural gas

## Abstract

**Background:**

One of the major areas for increasing the use of renewable energy is in traffic fuels e.g. bio-based fuels in diesel engines especially in commuter traffic. Exhaust emissions from fossil diesel fuelled engines are known to cause adverse effects on human health, but there is very limited information available on how the new renewable fuels may change the harmfulness of the emissions, especially particles (PM). We evaluated the PM emissions from a heavy-duty EURO IV diesel engine powered by three different fuels; the toxicological properties of the emitted PM were investigated. Conventional diesel fuel (EN590) and two biodiesels were used − rapeseed methyl ester (RME, EN14214) and hydrotreated vegetable oil (HVO) either as such or as 30% blends with EN590. EN590 and 100% HVO were also operated with or without an oxidative catalyst (DOC + POC). A bus powered by compressed natural gas (CNG) was included for comparison with the liquid fuels. However, the results from CNG powered bus cannot be directly compared to the other situations in this study.

**Results:**

High volume PM samples were collected on PTFE filters from a constant volume dilution tunnel. The PM mass emission with HVO was smaller and with RME larger than that with EN590, but both biofuels produced lower PAH contents in emission PM. The DOC + POC catalyst greatly reduced the PM emission and PAH content in PM with both HVO and EN590. Dose-dependent TNFα and MIP-2 responses to all PM samples were mostly at the low or moderate level after 24-hour exposure in a mouse macrophage cell line RAW 264.7. Emission PM from situations with the smallest mass emissions (HVO + cat and CNG) displayed the strongest potency in MIP-2 production. The catalyst slightly decreased the PM-induced TNFα responses and somewhat increased the MIP-2 responses with HVO fuel. Emission PM with EN590 and with 30% HVO blended in EN590 induced the strongest genotoxic responses, which were significantly greater than those with EN590 + cat or 100% HVO. The emission PM sample from the CNG bus possessed the weakest genotoxic potency but had the strongest oxidative potency of all the fuel and catalyst combinations. The use of 100% HVO fuel had slightly weaker and 100% RME somewhat stronger emission PM induced ROS production, when compared to EN590.

**Conclusions:**

The harmfulness of the exhaust emissions from vehicle engines cannot be determined merely on basis of the emitted PM mass. The study conditions and the engine type significantly affect the toxicity of the emitted particles. The selected fuels and DOC + POC catalyst affected the PM emission from the heavy EURO IV engine both qualitative and quantitative ways, which influenced their toxicological characteristics. The plain HVO fuel performed very well in emission reduction and in lowering the overall toxicity of emitted PM, but the 30% blend of HVO in EN590 was no better in this respect than the plain EN590. The HVO with a DOC + POC catalyst in the EURO IV engine, performed best with regard to changes in exhaust emissions. However some of the toxicological parameters were significantly increased even with these low emissions.

## Introduction

Current epidemiological data suggests that particles originating from traffic emissions are responsible for excess deaths and emergency hospital admissions in individuals living in vicinity of heavy traffic [1-3]. In addition, traffic-derived particulate air pollution has been associated with adverse effects on other cardiac [4,5] and respiratory health outcomes [6,7]. Most recently, convincing evidence has also been obtained about the carcinogenicity of diesel exhaust emissions [8,9]. Also the assessments of the International Agency for Research on Cancer (IARC) has recently changed the evaluation of diesel exhaust from probably carcinogenic to humans − Group 2A to carcinogenic to humans Group 1. However, even in this new evaluation the newest engine developments and new fuels are not included.

Oxidative stress and inflammation have been suggested as major toxicological mechanisms of the adverse health effects of particulate air pollution [10,11]. In toxicological set-ups it has been observed that diesel engine derived particles are able to cause acute systemic inflammatory effects in animals [12] and in human volunteers [13-15]. However, in the study of Tzamkiosis et al. [16], the influx of inflammatory cells and the increase in protein in bronchoalveolar lavage fluid of mice were rather modest when various diesel engines were studied. Similarly, in our previous *in vitro* study, we observed relatively small inflammatory potency by emission particles from Euro II diesel engine that was operated with the same fuels as the Euro IV engine in the present study [17].

The adverse health effects of urban air particles depend on many factors including particle size, surface area, number distribution, oxidative properties and chemical composition [18]. In the review of Hesterberg et al. [19], it was recommended that the safety evaluations of diesel engine emissions should be reassessed, because the properties of engines and fuels have improved significantly and the emissions are constantly changing. In particular, the PAH emissions have decreased when new engine technologies and low-sulfur diesel fuels with low levels of aromatics have been introduced [19]. The overall effects of new biofuels on the cascade of engine emissions and health-related toxicological parameters, however, are largely unknown [20]. Thus, the toxicological effects need to be characterized in greater detail, especially when a variety biofuels are already being introduced into wider use in heavy-duty diesel engines such as those installed in buses and trucks in cities.

It has been shown that biodiesels reduceparticulate and PAH mass emissions [21-23]. Some increases in NO_x_[23,24] and carbonyl [22] have been observed when fatty acid methyl ester (FAME)-biodiesel has been compared to conventional diesel fuel. The use of FAME has decreased oxidative activity [25] and cytotoxicity [23] as well as mutagenicity of the emission particles [23,26], but also increased bacterial mutagenicity has been reported with rapeseed methyl ester (RME) compared to natural gas derived synthetic fuel (gas-to-liquid, GTL) and conventional fossil diesel.

One proposed measure to achieve a reduction of particulate emissions in traffic has been the introduction of compressed natural gas (CNG) powered buses in the commuter traffic. The particulate emissions from these vehicles are lower than those from similar vehicles powered by conventional diesel fuels. Moreover, the emitted particles from CNG powered engines have been significantly less toxic [27,28] than those from liquid fuel powered engines. Therefore, one CNG powered bus was also included as a comparison to the present study.

Our aim was to examine the differences in urban traffic bus emissions, especially in toxicity between emission particles derived from a Euro IV heavy-duty diesel engine powered by European conventional, low-sulfur diesel (EN590) as well as by two different biodiesel fuels and their blends in EN590. The biodiesel fuels were rapeseed methyl ester (RME) and a more advanced hydrotreated vegetable oil (HVO). In addition, 30% blends of the biobased fuels in EN590 were tested. A new oxidation catalyst was employed in the study with EN590 and HVO. In the comparison with liquid fuels, the emitted particles from a compressed natural gas (CNG) powered bus were also studied.

The cellular parameters that were investigated represent the mechanisms by which particulate air pollution is believed to induce or exacerbate respiratory and cardiovascular diseases. These included production of inflammatory mediators (TNFα and MIP-2), cytotoxicity (MTT, cell cycle, apoptosis, necrosis), genotoxicity (Comet assay) and formation of reactive oxygen species (ROS). Macrophage cell line (RAW 264.7) was exposed to several doses of emission particles in order to examine their potency to induce toxicological responses. Chemical characteristics of the particulate samples were compared to their induced toxicological responses.

## Material and methods

### Engines and fuels

In the present study, the differences in emission between the liquid fuels were tested using a heavy-duty diesel engine that was attached to an engine dynamometer and the emission samples from compressed natural gas were collected from a bus that was driven on a chassis dynamometer. Thus, the results from CNG-powered bus cannot directly be compared to those gained from the engine dynamometer tests of different fuels. The engine that was used, was a direct injection, turbocharged and intercooled Scania of the model year 2005.It fulfilled the EURO IV emission class requirements and it had exhaust gas recirculation system. The volume of the six-cylinder engine was 11.7 liters and it had a maximum power of 310 kW and a maximum torque of 2100 Nm.

The compressed natural gas (CNG) powered bus for the comparison of the liquid diesel fuels was taken for the dynamometer tests directly from its duties in commuter traffic. The bus had a stoichiometric 11.9 liter, six cylinder engine. The bus was a year 2008 model and it had 206 000 driven kilometers. The CNG bus filled the requirements for the EEV (Enhanced environmentally friendly vehicle) emission class, in which the particle mass emissions are between Euro V and VI standards.

Both the diesel powered engine and the CNG powered bus were operated using a Braunschweig cycle that is one of the most common test cycles for buses. The cycle has a rather low overall load, which may have affected some of the emission parameters. The Braunschweig cycle is originally designed for chassis dynamometers, but it was in this case operated with engine dynamometer. The Braunschweig cycle describes better the driving conditions of the urban commuter traffic buses with multiple stops and low load conditions than the European transient cycle (ETS) that is used for the measurements when classifying the EURO standard emissions.

The cycle was converted to transient engine dynamometer by first driving a Scania bus on the chassis dynamometer and then converting the power and torque profiles to engine dynamometer.

Three different low sulfur fuels were included in the present study. The conventional diesel fuel was summer grade EN590 that had low sulfur (8 mg/kg) and PAH (1%) contents. RME (EN 14214) represented an older generation, esterified biofuel and had about the same sulfur content as EN590 and it contained no PAH compounds. RME was the only fuel containing oxygen. Due to its properties, only 7% addition of the RME is currently allowed to the diesel fuel that is sold in the European Union. In this study the hydro-treated vegetable oil (HVO) represented a new generation paraffinic biofuel. HVO had a lower sulfur content (5 mg/kg) and a much lower PAH content (0.01%) than EN590 and RME. The density of HVO was somewhat lower than those of RME and EN590 but its energy content per mass of fuel was higher as can be seen from the detailed fuels analysis [29].

The fuels were tested in the following combinations: EN590, HVO and RME as such (100%); 30/70% blend of HVO/EN590 (indicated as HVO 30%) and 30/70% blend of RME/EN590 (RME 30%). Moreover, a combined diesel oxidation catalyst (DOC) with particle oxidation catalyst (POC) was employed in the engine dynamometer tests together with 100% EN590 and 100% HVO. POC is a catalyst that with its substrates traps solid soot particles. POC should be used in combination with DOC since the latter produces NO_2_ for burning the soot collected by POC. [29,30].

### Sampling of the emitted particles

The particulate samples for toxicological experiments were collected from a constant volume dilution tunnel on two parallel filters (Fluoropore FSLW14200, PTFE 142 mm, Millipore) using a high volume sampler (800 liters per minute). Driving cycles and particulate collections were repeated with the same fuel several times to new filter sets to ensure that a sufficiently large sample mass would be obtained in order to complete the toxicological and chemical analyses. Blank control filters were treated similarly to the actual used filters except for sampling from the exhaust gas.

### Sample preparation for chemical analysis and cell studies

The filters were washed with methanol, dried and weighed prior to issuing them for the samplings. The filters were allowed to stabilize to room temperature overnight before weighing in an analytical balance (Mettler Toledo XP105DR) with a build in electrostatic charge remover. After the sample collection, the weighing procedure was repeated to obtain the collected mass. In the weighing room, temperature, relative humidity and air pressure were monitored, and the control weights as well as used reference filters were weighed. One more weighing for the selected filters was performed after the sample extraction procedure to obtain the extraction efficiency of the particulate mass [31].

Particulate samples were prepared for the cell experiments using previously validated procedures [32,33]. Briefly, the sampled PTFE filters were cut into pieces and placed in 50 ml glass tubes that were then filled with methanol. The samples were extracted 2 × 30 min in an ultrasonic water bath at a temperature below 35°C. The particulate samples were combined from extracts of each filter derived from collections of emission particles in connection to the use of each fuel. These methanol extracts were concentrated in a rotary evaporator to a small volume and the suspension was divided into glass tubes in aliquots based on the weighed mass. Finally, these concentrated suspensions, were dried in glass tubes under a flow of nitrogen gas (99.5%) and thereafter stored at −20°C.

It cannot be overruled that the extraction conditions would have led to uneven extraction efficiencies of different groups of components. The emission particles may contain different components depending on the fuel used in the engine. How these different compositions will suspend/extract to methanol is unclear. However, methanol was used as a compromise, because it has very good extraction/suspension efficiency from the filters by mass. Moreover, it has performed well in our previous studies with urban air particles [33] but it has also been tested with diesel exhaust particles [32].

### Chemical characterization of the collected particles

Concentrations of major inorganic ions (chloride, nitrate, sulphate, sodium, ammonium, potassium, magnesium, and calcium) were measured using two Dionex ICS-2000 ion chromatography systems. This method is described earlier in full details by Teinilä et al. [34].

In the trace element analysis two different sample pre-treatment procedures were used. A part of samples were treated with 0.08 M nitric acid and left for 30 min in ultrasonic bath for the determination of the easily soluble fraction. To achieve a more exhaustive digestion, part of the samples were digested with a solution of HF and HNO3 in an ultrasonic bath. Twelve different trace elements (Al, As, Cd, Co, Cr, Cu, Pb, Mn, Ni, Fe, Zn and V) were analyzed with an ICP-MS (Perkin Elmer Sciex 129 Elan 6000), using Rh as an internal standard [35].

A total of 34 PAH compounds were analyzed using a gas-chromatograph mass-spectrometer single ion monitoring technique (GCMS-SIM; HP 5890 GC, equipped with a HP 5970B Series Mass Selective Detector, Agilent Technologies, Germany) after extraction of the particulate samples with dichloromethane [36]. The sum of known genotoxic PAH compounds in particulate samples was calculated on the basis of the WHO-IPCS criteria [37].

The chemistry data from the high-volume particulate samples were complemented with the thermal optical analysis of elemental carbon (EC) and organic carbon (OC) from parallel low volume particulate samples. The OC concentration was multiplied by 1.6 to obtain particulate organic matter (POM) containing – in addition to the measured carbon - anticipated amounts of hydrogen, oxygen and nitrogen [38].

### Cell culture

A mouse macrophage cell line RAW264.7 was cultured in RPMI 1640 cell culture medium that was supplemented with 10% heat-inactivated fetal bovine serum (FBS), 1% L-glutamine and 1% penicillin streptomycin (Gibco BRL, Paisley, UK). The cells were grown in the culture bottles at +37°C in a 5% CO_2_ atmosphere. In the experiments, the cell suspension was diluted to 5x10^5^ cells ml^-1^ and cultured on 6-well plates (Costar, Corning, NY, USA) for 24 hours to allow the cells to adhere and divide. One hour before the experiments fresh medium (+37°C) was changed on the cells and they were allowed to stabilize.

### Experimental setup for toxicological studies

Before the cell exposure, sample tubes were treated in an ultrasonic water bath for 30 minutes to suspend the particulate samples into pyrogen free water (Sigma W1503, St. Louis, MO, USA) at a concentration of 5 mg ml^-1^. The blank samples were treated similarly to the actual particulate samples. A small, non-toxic amount of dimethylsulfoxide (DMSO 0.3% v/v at dose 150 μg ml^-1^) was used to facilitate suspension of the collected particulate mass into water. Cells were exposed to four doses (15, 50, 150 and 300 μg ml^-1^) of each particulate sample for 24 hours in three independent experiments that were conducted in duplicate. Untreated cells as well as blank samples in a volume corresponding to the particulate mass dose of the actual samples150 μg ml^-1^ were used in the experiments as controls to the exposed cells.

After the 24-hour exposure, the cells were resuspended into cell culture medium by scraping them from the bottom of the wells. A total of 2 × 100 μl of cell suspension from each well was taken for the MTT analysis of cytotoxicity. The remaining cell suspension was centrifuged and the supernatants were stored at −80°C for subsequent cytokine analysis [32]. The cells were washed, suspended in phosphate buffered saline (PBS) and aliquoted into two portions. One portion was fixed in 70% (v/v) ethanol for subsequent propidium iodide staining [39] and the other was used in the detection of cell membrane permeability/necrosis. The parallel duplicate well was treated similarly, but it was used for the analysis of genotoxicity. One more well with the cells was grown for detection of reactive oxygen species.

### Analyses of inflammation

The proinflammatory cytokine tumor necrosis factor alpha (TNFα) and the chemokine, macrophage inflammatory protein 2 (MIP-2) were immunochemically analyzed from the cell culture medium, using commercial enzyme linked immunosorbent assay (ELISA) kits (R&D Systems, Minneapolis, MN, USA) as earlier described in detail [32]. Blank samples were included in all the experiments in order to rule out methodological artifacts. LPS was used as a positive control for cytokine analyses to ensure the methodological reliability.

### Analyses of cytotoxicity

#### MTT test

The viability of the macrophages was detected with the (3-(4,5-dimethylthiazolyl-2)-2,5-diphenyltetrazolium bromide) (MTT)-test on 96-well plates. The absorbance was detected with the plate reader and the viability was calculated as a percentage from corresponding readings of the control cells [32]. Any potential interference of the particulate samples with the method was also tested and ruled out. Diesel reference material was used as a methodological control.

#### Cell membrane permeability

The amount of propidium iodide (PI)-positive cells with a lowered cell membrane potential was detected by flow cytometry [17]. These cells can be considered as necrotic or late apoptotic in the analysis. A total of 10,000 cells per sample were analyzed with a flow cytometer using Summit software version 4.3. Diesel reference material was used in experiment to ensure that the cell model was giving repeatable results.

#### Apoptosis and cell cycle

The cellular DNA content was analyzed by PI staining of permeabilized cells in a flow cytometer. This method provides information about the cell cycle and apoptotic cells can be identified as the cells containing fragmented DNA (number of hypodiploid cells as in sub G_1_ peak) [40,41]. The feasibility of this indirect method that has been successfully used in some previous studies to assess the proportion of apoptotic cells has also been confirmed in RAW264.7 macrophages, as an association between the expression of caspase enzymes and the subG1 proportion of the cells [39].

A total of 10,000 cells wereanalyzed per sample at an emission wavelength of 613 ± 20 nm by flow cytometer. [39]. Possible interference of the method with particles was also studied and proved to be negligible after adjustment of the gates in the analysis. Etoposide was used as a positive control to induce apoptosis in the cells.

### Analysis of genotoxicity

Deoxyribonucleic acid (DNA) damage was detected in the alkaline Single cell gel/Comet assay that was originally described by Singh et al. [42] and later slightly modified by Jalava et al. [17]. In the comet assay, DNA strand breaks and those associated with incomplete excision repair sites can be detected, and therefore the potential mutagenicity and carcinogenicity can be estimated. The nuclei were analyzed in ethidium bromide-stained (100 cells per dose) using an image analysis system (Comet assay IV, Perceptive Instruments Ltd., Suffolk, UK). The Olive tail moment ((tail mean –head mean) x tail%DNA/100) was the parameter used in the statistical analysis. Methylmethanesulfonate and benzo [a] pyrene were used as positive controls in the analyses of genotoxicity.

### Analysis of reactive oxygen species (ROS)

The intracellular accumulation of ROS was measured by flow cytometry using the fluorescent probe, 2´,7´-dichlorodihydrofluorescein diacetate (H_2_DCFDA; Molecular Probes). In the presence of H_2_O_2_ or other peroxides, H_2_DCFDA is oxidized to the fluorescent product, 2´,7´-dichlorodihydrofluorescein (DCF), indicative of the intracellular amount of peroxides. In the present study, during the last 30 min of the incubation, RAW264.7 cells were loaded with 1 μM H_2_DCFDA in phosphate-buffered saline (PBS; Gibco). The cells on six well plates were washed with PBS and thereafter scraped from the bottoms of the wells. The cell suspension was centrifuged (5 min at 370 × g), and washed once more with 1 ml of PBS. The pellet was suspended in 1 ml of PBS and the fluorescence signal of DCF positive cells was analyzed in a flow cytometer (CyAn ADP; Beckman-Coulter, CO, USA). A total of 10,000 cells were analyzed per sample using Summit software version 4.3. (Beckman-Coulter). Menadione was used as a positive control for these analyses.

### Statistical analysis

Levene’s test for equality of variances was used for all the samples before using analysis of variance (ANOVA). The various responses of macrophages to particulate exposures were tested against the corresponding blanks as well as with regard to particle dose. Paired comparisons between the different particulate doses were made by Dunnett’s test. ANOVA and Tukey´s test were used for comparison of the differences in responses in connection to different fuels and the catalyst. The cell-cycle, PI-exclusion, ROS and comet assay results were tested with the non-parametric Kruskall-Wallis test. All the differences were regarded as statistically significant at p < 0.05. The data were analyzed using the SPSS statistics version 17.0 (SPSS Inc. Chicago, IL, USA).

## Results and discussion

We observed considerable differences in the toxic potencies between emission PM collected from the EURO IV engine powered by different fuel and catalytic converter combinations. In most parameters, the potencies of emission PM with the use of HVO were weaker than those obtained with EN590and the same trend was observed in fewer parameters with RME. The DOC + POC catalytic converter decreased substantially PM mass emissions with EN590 and HVO, and in mostcases, also the toxic activities of the PM were reduced. The CNG powered bus that was included in the study as a comparisonwas anticipated to have the lowest PM mass emission, but when investigated on equal mass doses in various toxicological setups, these emission PM samples showed highly variable toxic properties: the strongest oxidative and MIP-2 inducing potency but the weakest genotoxic potency of all the tested fuel and catalyst combinations. However, these results cannot be directly compared due to the chassis dynamometer used with the CNG-bus.

### Emissions

Emission data is presented earlier in details by Murtonen et al. [29]. The PM mass emissions from the EURO IV engine did not depend exclusively on the fuel, because the DOC + POC catalytic converter had also a large effect on the emission with EN590 and HVO. The finding of HVO is in agreement with previous studies, where the paraffinic fuel has decreased emissions of nitrous oxides (NO_x_), PM, carbon monoxide (CO) and hydrocarbons (HC) from various engines [29]. From the present engine, the NO_x_ emission was slightly larger with HVO than with EN590. This may be due to the lower density of the HVO fuel and its effect on the emission gas recirculation as seen also by Clark et al. [43]. Usually the NOx emissions are raised, but the particulate emissions decreased when methyl ester fuels are used [44]. However, now a contradictory finding to many other studies was observed. Thisdiscrepancy could be well explained the high proportion of soluble organic fraction (SOF) due to relatively low load imposed during the Braunschweig test cycle in the present study [45,46]. In this case, over 70% of the PM emission with RME consisted of the SOF, whereas with HVO and EN590, the corresponding fractions were less than 40%. This alone was explaining the higher PM mass emissions for RME in these study conditions and for the engine used in the present study. It is possible that the emissions could have been somewhat smaller, if the engine parameters had been separately readjusted for each individual fuel.

PM samples, collected for toxicological analyses from the EURO IV engine operated with different fuels were as follows: EN590 22.2 mg/kWh, HVO 12.8 mg/kWh and RME 46.7 mg/kWh. Addition of biodiesel fuels to EN590 also affected the mass emissions. The results after different fuel blends were: 23.7 mg/kWh with 30% HVO and 35 mg/kWh with 30% RME. Catalyst reduced the PM emissions to 8.7 mg/kWh in case of EN590 and to 4.4 mg/kWh when HVO was used. Therefore also the collection times for the small emissions were longer.

### Chemical composition of the particulate samples

We have previously shown that the chemical characteristics of urban air fine PM have a significant role in mediating the toxicological effects both in cells [47] and mouse lung [48]. It is also known that the quality and quantity of PM emissions from different engines can be very different, which subsequently alters their toxicological characteristics (e.g. 16).

#### Concentrations of the PAH compounds in the emissions

The results in chemical analyses are presented in Table 1. The largest total PAH emission (820 ng mg^-1^ PM) was measured from the emission particles with EN590 and the PAH contents were only slightly reduced with 30% blends of either HVO (809 ng mg^-1^) or RME (710 ng mg^-1^). When the plain biodiesel fuels were used, the total PAH content of the emitted PM was 613 ng mg^-1^with HVO and 491 ng/mg with RME. The catalyst reduced the total PAH content with EN590 (115 ng mg^-1^) to a clearly lower level than with HVO (205 ng mg^-1^). The total PAH emission from the CNG bus was the lowest (61.7 ng mg^-1^) of all fuel and catalyst combinations. Thus different conditions significantly affect the PAH compositions of the emitted particulate matter.

**Table 1:** **Chemical constituents (ng/mg PMmass) in particles emittedfrom the heavy-duty dieselengine operated with EN590,HVO (30% and 100%),or RME (30% and100%)**

**Chemical composition (ng mg**^**-1**^ **PM)**	**EN590**	**EN590 cat**	**30% HVO**	**100% HVO**	**HVO cat**	**30% RME**	**100% RME**	**CNG**
*Organic constituents*								
Sum of PAH compounds	820	115	809	613	205	710	491	61.7
Sum of genotoxic PAHs*	380	35.2	381	257	66.3	422	340	26.4
*Inorganic ions*								
Na^+^	690	709	352	465	1530	260	370	2390
NH_4_^+^	137	139	356	163	249	372	250	199
K^+^	23.9	29.6	19.0	17.1	34.3	51.7	145	116
Cl^-^	113	133	48	138	210	32.7	49.2	459
SO_4_^2-^	143	156	170	145	212	115	46	777
NO^3-^	1090	1150	451	734	2390	449	712	1360
*Elemental composition*								
Cd	0.2	0.1	0.4	0.3	0.6	1.0	0.7	1.1
Co	23.8	2.1	27.5	9.8	22.6	81.6	10.5	313
Cr	36.0	32.2	32.8	33.6	45.6	43.7	43.1	226
Cu	96.4	51.5	83.0	66.9	74.6	120	61.7	214
Fe	610	274	868	424	855	2490	331	1690
Mn	62.9	24.5	82.9	23.4	141	658	19.4	94.4
Ni	19.1	8.5	23.8	15.7	28.3	35.8	13.7	267
Pb	3.6	2.2	4.1	6.8	11.0	11.2	5.0	19.8
V	1.8	1.0	2.0	1.6	4.8	3.0	1.0	26.9
Zn	2340	1190	3350	2070	716	2390	671	2280

* GenotoxicPAHs as defined by WHO/IPCS (1998).

EN590 and 100% HVO were used with and without DOC + POC catalytic converter.

The portion of known genotoxic PAH compounds was somewhat different from that of total PAHs. PM mass from the engine operated with EN590 had the largest content of genotoxic PAH compounds (380 ng mg^-1^), followed by RME (340 ng mg^-1^) and HVO (257 ng mg^-1^). HVO 30% had no difference from EN590 but RME 30% had a slightly higher PAH content of the emission PM. The DOC + POC catalyst reduced by 90% the proportion of known genotoxic PAHs in the PM mass emitted with EN590 fuel and by 75% that emitted with HVO. As a comparison, the PM mass emitted from the CNG bus contained the smallest amount of genotoxic PAH compounds (Table 1). The current PAH emission results are different from those seen in our previous study on which examined a small industrial EURO II diesel engine, as no genotoxic PAHs were detected in the latter samples [17]. This discrepancy can be explained by various factors; the engines were different, they were presenting different sizes, technologies and emission classifications. Moreover, in the study with Euro II engine, a combination of steady state conditions was used instead of transient cycle. However, the results clearly show that various parameters, including engine type and test cycle can affect the harmfulness of the emission particles even if the same fuels are used. One factor explaining differences would be the nitrated PAHs, but they were unfortunately not measured in the present study.

#### Concentrations of inorganic ions in the emissions

The lowest PM mass emissions in the cases of HVO + cat and CNG were associated with the largest contents of Na^+^ in the emitted PM. The level of ammonium in the emitted PM mass was otherwise rather similar with all of the fuel and catalyst combinations, with the exception that HVO 30% and RME 30% increased its amount. Potassium was enriched in the emission PM mass, when RME and CNG were used as fuel. Sulfate emissions were generally at a low level due to the low sulfur content of the fuels. The minimal PM mass emitted from CNG bus contained a much higher amount of sulfate than the PM samples produced with other fuel and catalyst combinations. In the CNG-powered engine, there is a higher possibility that these emissions are derived from lubricant oil as compared with the other engine in this study.

#### Metal concentrations in the emissions

Zinc and iron dominated the metal contents of the PM mass emitted with all fuel and catalyst combinations from the EURO IV engine. The largest total metals per emitted PM mass were seen with the low-emission CNG bus. The metals are probably derived from the engine wear and lubrication oils which enrich due to longer sample collection time of the low emissions. One interesting feature was that RME 30% - and tomuch lesser extent HVO 30% - increased concentrations of most metals in the emission PM as compared to EN590. The larger metal concentrations in the RME fuel might explain this and the larger fuel consumption would increase this difference. For HVO this cannot at least be explained by enrichment of the metals, since the total emitted PM mass was rather similar between the fuels.

In the case of EN590, the catalyst reduced the contents of all metals in the emitted PM mass. The situation was the opposite when the catalyst was employed with HVO, except for zinc. At the same time, the PM mass emissions decreased, but the difference cannot solely be explained by that change. From the studied fuels, the smallest contents of metals in the emitted PM mass were observed with RME. This is an opposite finding to our previous study with a small EURO II diesel engine [17]. It is also otherwise surprising since the RME fuel contains the largest metal concentrations among the studied fuels. It may be possible that the other compounds in the emissions bind the metals and they are not equally well extracted from the samples.

It has been noted in previous studies that aftertreatment of engine exhaust may change the oxidative potential of emitted PM [49] and that transition metals in the PM samples have in some cases enhanced the oxidative potential [50]. However, effective particle traps have diminished these effects in addition to reducing the PM mass emission [51]. It is noteworthy that methyl ester fuels cannot be used as high ratios in the engines when modern particle reduction aftertreatment technologies are used. The metals and other impurities in the fuels may lead to faster reduction of catalyst efficiency.

### Effects of fuel and catalyst combinations on toxicological responses

#### Inflammatory mediators

The emission PM samples from EURO IV engine powered by different fuel and catalyst combinations induced only low to moderate inflammatory responses. Nonetheless even this level of inflammation should not be ignored due to its important role in PM involvement in cardio-respiratory diseases [10]. It has also been observed that diesel exhaust particles can induce systemic inflammation in rats [12] and increased inflammatory cell and adhesion molecule expressions in human exposures [15]. However, in a recent review by Ghio et al. [52] it has been concluded that inflammation by diesel particle exposure is obvious only at large concentrations. They suggest that the gaseous emissions may also be behind the adverse effects. This is one reason why in the future it is essential to develop methods where also gaseous composition of the emissions can be studied in *in vitro* systems in parallel to particles.

#### TNFα production

**Figure 1:**
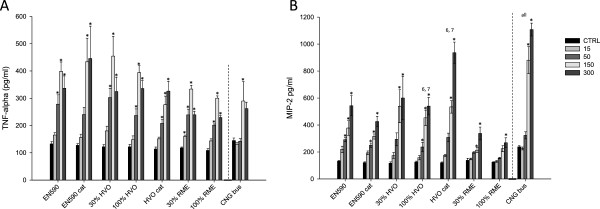
**A. Tumor necrosis factor alpha (TNFα).** and **B.** macrophage inflammatory protein 2 (MIP-2) (pgml^-1^) after a 24-h exposure of mouse macrophage cells RAW 264.7 to emission particle (PM) samples from EURO IV engine that was run with different fuel/DOC + POC catalytic converter combinations. Bars show control level and responses to four PM doses (15, 50, 150 and 300 μg/ml) and whiskers show the standard error of the mean (SEM). Asterisks indicate statistically significant differences from the control (ANOVA and Dunnett’s test; p < 0.05). Numbers refer to a significantly larger response (ANOVA and Tukey’s test; p < 0.05) compared to the indicated other fuel/catalytic converter combination: 1) EN590, 2) EN590 + cat, 3) 30% HVO, 4) 100% HVO, 5) HVO + cat 6) 30% RME, 7) 100% RME, and 8) CNG bus.

The TNFα production after 24-hour exposure of macrophages to the PM samples is shown in Figure 1A. All the samples, at least at some PM doses, increased statistically significantly the TNFα levels compared to the control. However, there were no statistical differences between the PM samples with different fuel and catalyst combinations and no sample emerged as more potent. Instead, in the chemokine response there were some differences between the samples.

#### MIP-2 production

Figure 1B shows the chemokine MIP-2 concentrations induced by the emission PM samples. All samples caused statistically significant, dose-dependent increases in the MIP-2 concentration. Overall, the levels of induced TNFα and MIP-2 concentrations were lower than those measured in the same cell line after exposure to emission PM from older technology diesel engine powered by the same three fuels [17]. Now when RME 30% and RME were used as fuels, the MIP-2 productions after exposure to emission PM were among the lowest of all samples. The emission PM samples from the engine powered by the HVO and catalyst combinationhad the highest MIP-2 responses among the EURO IV samples. As a comparison, the response to the CNG bus emission PM at the highest dose level was larger than the corresponding responses to any other PM sample. These differences indicated that the chemical composition of emission PM was associated with their toxicological responses. In the correlation analyses most of the associations were weak or inconsistent; the table of these analyses is presented as Additional file 1.

Previously, it has been shown in mice that emission PM from older technology diesel engines may induce lower inflammatory responses than those from newer engines [16]. Also operating conditions have affected the inflammatory responses to emission PM, higher engine loads inducing larger responses [53]. The reason for low inflammatory effects may be the suppression of immune system by the emission PM from diesel engines and other combustion processes (e.g. 47, 48). It is possible that different expressions of MIP-2 and TNFα in the present study are due to different pathways of the cytokines being activated as seen previously with IL-8, IL-6 and COX-2 in human bronchial epithelial cells [54]. Overall, the inflammatory parameters induced by emission PM showed greater variation between the three fuels in the present study than in our previous study with the smaller EURO II diesel engine [17]. This indicates that the role of the engine type and operational parameters may in fact be larger than that of the fuels in determining the emission PM induced inflammation. In previous studies, emissions of methyl ester biodiesel operated engines have increased even more intensive inflammatory responses in the mouse blood and lungs [55] but have shown less inflammatory activity in endothelial cells [56]. There are no previous studies where the effects of emission PM from HVO fuel powered modern diesel engine have been examined through such diverse toxicological pathways.

#### Cytotoxicity

**Figure 2:**
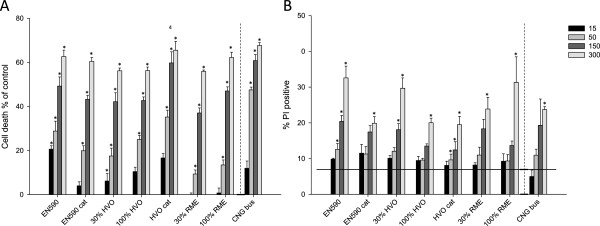
**A. Cell death assessed with the MTT test after a 24-h exposure of RAW 264.7 macrophages to emission PM samples from EURO IV engine run with different fuel/DOC   + POC catalytic converter combinations.** Bars represent control level and responses to four PM doses (15, 50, 150 and 300μgml^-1^) and whiskers show the standard error of the mean (SEM). Asterisks indicate statistical significant differences from the control (ANOVA and Dunnett’s test; p < 0.05). **B**. Cell death (mostly necrosis) detected with PI-exclusion method in the same exposure of RAW 264.7 cells as in panel **A**. Asterisks indicate statistically significant differences from the control level (Kruskall-Wallis; p < 0.05). Numbers refer to a significantly larger MTT-test response (ANOVA and Tukey’s test; p < 0.05) or PI-exclusion test response (Dunnett’s C test; p < 0.05) compared to the indicated fuel/catalytic converter combination: 1) EN590, 2) EN590 + cat, 3) 30% HVO, 4) 100% HVO, 5) HVO + cat 6) 30% RME, 7) 100% RME, and 8) CNG bus.

The results of acute cytotoxicity as assayed with MTT-test are shown in Figure 2A. All the samples evoked dose-dependent increases in the cell death. The emission PM samples with EN590, HVO + cat and CNG-bus were statistically significantly cytotoxic even at the lowest dose, when compared to controls. However, there was only one statistical difference that was observed between the samples;HVO and HVO + cat. Cell death was also detected in the PI-exclusion test as shown in Figure 2B. All the PM samples, most often at the two largest doses, induced significantly increased responses when compared to control. The cell membrane permeability which is indicative of necrotic or late apoptotic cells, was greatest with the emission PM samples with EN590, HVO 30% and RME (100%). It has been observed previously that diesel exhaust PM can induce necrotic cell death in human bronchial epithelial cells [54]. In the study of Kooter et al. [23], the emission PM from engine powered by 100% methyl ester biodiesel have been significantly more cytotoxic in the lactate dehydrogenase (LDH) test than those from engines using conventional diesel fuel. Moreover, in the study of Bunger et al. [26] emission PM with RME have been more toxic to fibroblasts than PM with conventional diesel. Finally, there are indications that biodiesel blends can produce emission PM, which have higher cytotoxic potential than conventional diesel fuel [57].

**Figure 3:**
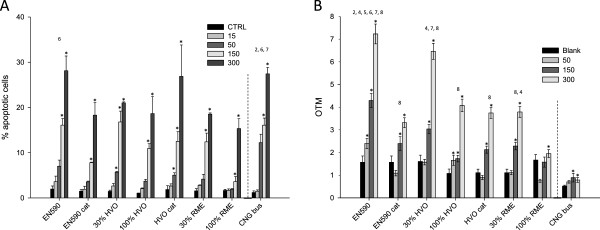
**A. Apoptosis assessed with flow cytometric detection of permeabilized cells after a 24-h exposure of RAW 264.7 macrophages to emission PM samples from EURO IV engine run withdifferent fuel/DOC + POC catalytic converter combinations.** And **B**. Genotoxicity assessed with the comet assay. Bars present blank levels and responses to three PM doses (50, 150 and 300 μg ml^-1^) and whiskers show the standard error of the mean (SEM). Asterisks indicate statistically significant differences from the blank control (Kruskall Wallis; p < 0.05). Numbers refer to a significantly larger response (Dunnett’s C test; p < 0.05) compared to the indicated fuel/catalytic converter combination: 1) EN590, 2) EN590 + cat, 3) 30% HVO, 4) 100% HVO, 5) HVO + cat 6) 30% RME, 7) 100% RME, and 8) CNG bus.

Exposure to diesel exhaust particles has increased apoptosis [58,59]. In the present study, programmed cell death was analyzed in a flow cytometric assay of PI stained fixed cells (Figure 3A), where all PM samples evoked dose-dependent, statistically significantly increased responses compared to control. There were no significant differences between the PM samples with different fuel and catalyst combinations with regard to their ability to cause apoptosis. However, the responses for EN590, HVO + cat and CNG bus were somewhat higher with the largest PM doses. Generally, the PM samples evoked similar or slightly smaller apoptotic responses than seen in our previous study with the small EURO II diesel engine [17], but this time, the variation was larger between the fuel and catalyst combinations. This confirms that both engine and fuel can make major contributions to the emission properties as previously shown by Kennedy et al. [60]. According to our present results, also exhaust after-treatment should probably be added to the list.

#### Genotoxicity

We have very recently received new evidence that long-term exposures of miners to diesel exhaust has strongly increased their risk of lung cancer and some other cancers [8,9]. Also IARC changed very recently the classification of diesel exhaust to be carcinogenic to humans. However, emissions from the diesel engines at least in road traffic have changed significantly during the past decade, when new technologies, including cleaner engines, better fuels containing low levels of sulfur and aromatics, and effective emission after-treatment, have been introduced into new cars and are now in wider use [19]. Especially the PAH composition in particulate phase, which is known to cause cancer, has been drastically reduced in car emissions and urban road traffic.

The PM samples in the present study induced dose-dependent genotoxic responses in the comet assay similar to the PM samples from the small EURO II engine in our previous study [17]. However, the present results exhibited larger variations between the fuel and catalyst combinations, which made it possible to detect significant positive associations of the genotoxicity with the PAH and Zn content of the PM samples. The data for these correlations is presented in the Additional file 1. In the recent study of Hemmingsen et al., [56] the emission particles from the EURO 4 engine were more potent than those from the EURO 2 engine in their ability to evoke DNA damage. However, they studied light duty engines, while we used a modern EURO IV heavy diesel engine.

Emission particles from the engine powered by EN590 and HVO 30% were the most potent inducers of DNA damage in the comet assay, which was likely attributable to the high total PAH contents in their emission PM samples. Moreover, all PM samples, except that from the RME 100% sample showed statistically significantly greater genotoxicity than the least genotoxic CNG-bus derived PM sample. The DOC + POC catalytic converter reduced significantly the genotoxic potencies of PM mass with both EN590 and HVO, and this was most probably due to the greatly reduced PAH content in the PM mass.

From the liquid fuels, RME produced the least potent PM samples in inducing DNA damage in the 264.7 macrophages. This result is supported by recent studies (e.g. 56). In contrast, the mutagenic potency of emission PM from EURO III truck engineshas been greater with methyl ester biodiesel than with conventional fossil diesel at least in two studies [23,61]. In the latter study, the gas to liquid (GTL) fuel resembling newest biomass to liquid (BTL) fuels was equal to the normal EN590 diesel in the mutagenic activity of the emissions. Again, it is the engine type, which seems to be a major determinant of the emission PM induced responses and the effects of fuels can be applied only to one engine type. These present results indicate that PM emissions and their genotoxic potential from a large EURO IV engine can be reduced by using renewable fuels and that these reductions can be enhanced by the installation of a DOC + POC catalytic converter. The catalyst was not tested with RME, because their combined use is not an option in real life because the impurities of the RME even if it fulfills the standards may destroy the catalyst sooner than the other fuels when high blend ratios are used. The catalyst needs higher quality biodiesel fuels like HVO or BTL to remain operative over the longer term.

The emission PM sample from the CNG powered bus was the least potent inducer of genotoxicity, which was likely attributable to its very low PAH content. Previously, there have been indications that also the emission PM from CNG vehicles can induce mutagenic activity [28]. However, emission PM sample from CNG powered Euro II engine have had lower mutagenic activities than samples from liquid fuel in a similar engine in the study of Turio-Baldassarini et al. [27]. The bacterial mutagenic assay used in those studies does not fully compare to the DNA-damage observed in mammalian cells used in the present study. Moreover, the CNG bus used in our study met the tight EEV emission limits for both particles and NO_x_[29].

#### ROS production

There are previous indications that the after-treatment of emissions from heavy-duty diesel engines affects the oxidative activity of the emission PM [49]. Moreover, Cheung et al. [25] have shown that the oxidative stress potential by emission PM may be increased with biodiesel fuel when compared to conventional fossil diesel but per driven kilometers the oxidative potential of the biodiesel was smaller. Also Kooter et al. [23] have shown that the emission PM from biodiesel-powered engine can cause a smaller oxidant reaction in the DTT-assay than PM from conventional fossil diesel powered engine. Also in our data, the comparison of the results by output power would change the order of potency between the samples (Additional file 2).

**Figure 4:**
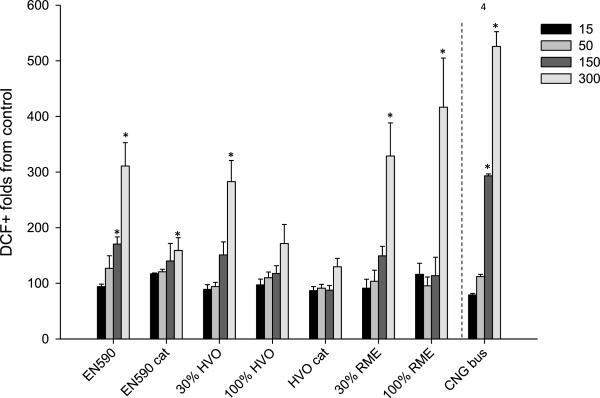
**Oxidative stress assessed with DCF assay, where intracellular peroxides can be identified from the cells emitting positive signals.** Bars represent responses to four PM doses (15 50, 150 and 300 μg ml^-1^) and whiskers show the standard error of the mean (SEM). Asterisks indicate statistically significance significant differences from the control level (Kruskall Wallis; p < 0.05). Numbers refer to a significantly larger response (Dunnett’s C test) compared to the indicated fuel/catalytic converter combination: 1) EN590, 2) EN590 + cat, 3) 30% HVO, 4) 100% HVO, 5) HVO + cat 6) 30% RME, 7) 100% RME and 8) CNG bus.

In the present study, the ROS production after the exposures of 264.7 macrophages to various emission PM samples is shown in Figure 4. All the samples except for those from HVO and HVO + cat induced statistically significantly increased ROS production at least at the highest PM doses. The PM sample from the CNG bus was the most potent in causing oxidative stress in macrophages on an equal mass basis, but the evoked response differed significantly only from that induced by the HVO derived PM sample.

The use of HVO with or without the catalyst showed weaker oxidative potential of emission PM, when compared to EN590 fuel. However, the catalyst reduced also the oxidative potency of emission PM derived from EN590 use. Our results on RME derived emission PM showed increased oxidative potential in the macrophages when compared to EN590 but only with the largest dose. This has some discrepancy with the previous studies [23,25,56], where the emission particles from RME powered engine caused weaker or similar ROS production when compared to standard diesel fuel. It may be that the unusual composition of our RME sample with high proportion of SOF has induced oxidation. The differences between the fuels were larger when the emission factor (mg PM/kWh) was taken into account (Additional file 2). There is also a possibility that the 24 hours time-point for the analyses of ROS could have been too late to compare the different situations. This issue has to be addressed in the forthcoming studies on the diesel exhaust particles.

There is very limited amount of information available on how hydro-treated vegetable oils affect the toxicity of engine emissions. In the present study with a heavy EURO IV engine, the HVO fuel consistently decreased the oxidation potential of the PM samples and the effect was even enhanced with the DOC + POC catalyst. This finding is opposite to the results from our previous study on the emission PM from a EURO II compliant engine using the same fuels [17]. The oxidative response to the CNG bus derived PM was the largest, which can be explained by the higher than average metal content in its emission PM.

## Conclusions

The harmfulness of the exhaust emissions from vehicle engines cannot be determined merely on the basis of emitted PM mass. The selected fuels and DOC + POC catalyst affected the PM emission from the heavy EURO IV engine both in qualitative and quantitative terms. However, the engine types as well as the test cycle load seem to affect the toxicity of emission PM to a significant degree which was in the present study seen as an unusually high SOF content of emissions when RME was used. Thus, the present results only apply in these study conditions.

The plain HVO fuel performed very well in emission reduction and in lowering the overall toxicity of emitted PM, but the 30% blend of HVO with EN590 was no better in this respect than plain EN590. The reasons for this failure remained unknown. HVO with the DOC + POC catalyst in the present engine was emitting very low levels of particulate mass. Even though some of the toxicological parameters were raised compared to the EN590 fuel, the low emission mass probably compensates this difference. CNG bus performed best with regard to exhaust emissions mass and when this low mass is considered, it probably reduces overall harmfulness of these emissions. However, these results cannot be directly compared to others since they derived from different engine and dynamometer. From the toxicological point of view the RME fuel generally decreased the toxicity compared to different fuels. However, the largely increased particulate mass counteracts this effect. (Additional file 2). Moreover, in the real life, only small proportion of fuel can be replaced with RME. One lesson to be learned from the present study was that even a low PM mass emissions might harbor a significant health-relevant toxic potential.

## Competing interests

The authors report no conflicting interests.

## Authors’ contributions

PIJ, MSH and AM wereresponsiblefor toxicologicalexperiments,includingcytotoxicity, inflammation and oxidativestress and for the extractions of the samplematerialfromfilters. PH and JM-P contributed to the toxicologicalexperiments with expertise in genotoxicity. PA-S and TM wereresponsible for design of engineexperiments and samplecollections for toxicological and chemicalanalyses. PY-P did the PAH analyses, whereasotherchemicalanalysesweredone in group of RH. RH, JJ, ROS and M-RH wereinvolved in the design and coordination of the study. PIJ was the corresponding author of the manuscript but all the authors contributed to the preparation of the manuscript. All authors read and approved the final manuscript.

## Supplementary Material

Additional file 1 Correlation coefficients between chemical composition and toxicological responses of Euro IV engine emissions. Click here for file

Additional file 2 Relative responses between the samples at dose 150 μg/ml as well as the results weighed with emission factor mg/kWh. Click here for file
