# Supplementary material for: Toxicological properties of emission particles from heavy duty engines powered by conventional and bio-based diesel fuels and compressed natural gas
Source: Part Fibre Toxicol. 2012 Sep 29;9:37. doi: 10.1186/1743-8977-9-37 (PMC3543388; doi:10.1186/1743-8977-9-37)
Supplement: Additional file 1 — Correlation coefficients between chemical composition and toxicological responses of Euro IV engine emissions. [file 1743-8977-9-37-S1.rtf]

 	 	 	TNFα	MIP-2	MTT	Apoptosis	PI-excl.	Genotoxicity	ROS	
Organic components								
Sum of PAH		-0.213	-0.228	0.142	-0.166	-0.177	0.69*	-0.221	
Sum of Genotoxic PAH	0.16	-0.474*	0.213	-0.412*	-0.258	0.524*	-0.365*	
Inorganic ions									
Na+			-0.047	0.547*	-0.247	0.588*	0.426*	-0.31	0.557*	
NH4+			-0.022	-0.328	0.195	-0.258	-0.344*	-0.19	-0.438*	
K+			-0.103	-0.324	-0.082	0.061	0.28	-0.571*	0.332*	
Cl-			-0.168	0.658*	-0.258	0.595*	0.266	-0.5*	0.345*	
SO42-			-0.023	0.703*	-0.119	0.385*	0.074	-0.143	0.201	
NO3-			0.03	0.495*	-0.191	0.583*	0.42*	-0.238	0.497*	
Elemental composition								
Cd			-0.146	-0.17	-0.068	0.115	0.133	-0.655*	0.133	
Co			-0.137	0.246	-0.145	0.111	-0.023	-0.06	0.185	
Cr			-0.187	0.162	-0.202	0.385	0.215	-0.595*	0.303	
Cu			-0.159	0.286	-0.198	0.169	0.037	-0.024	0.262	
Fe			-0.241	0.128	-0.011	-0.193	-0.319	-0.357	-0.267	
Mn			0.007	0.111	-0.015	-0.063	-0.037	0.119	0.07	
Ni			-0.157	0.327*	-0.146	0.182	-0.021	-0.214	0.136	
Pb			-0.327*	0.283	-0.193	0.244	-0.025	-0.699*	0.03	
V			-0.178	0.479*	-0.189	0.315	0.029	-0.295	0.166	
Zn	 	 	-0.009	0.131	0.007	-0.249	-0.257	0.524*	-0.104	
										
Additional file 1. Spearman's correlation coefficients between the chemical constituents of emitted PM and the toxicological responses. Asterisks indicate statistically significant correlation. The results from a dose 150μg/ml of all the responses in Euro IV engine dynamometer tests was used in the correlation analyses. 
