# Supplementary material for: Toxicological properties of emission particles from heavy duty engines powered by conventional and bio-based diesel fuels and compressed natural gas
Source: Part Fibre Toxicol. 2012 Sep 29;9:37. doi: 10.1186/1743-8977-9-37 (PMC3543388; doi:10.1186/1743-8977-9-37)
Supplement: Additional file 2 — Relative responses between the samples at dose 150 μg/ml as well as the results weighed with emission factor mg/kWh. [file 1743-8977-9-37-S2.rtf]

Additional File 2. The relative toxicological responses by the emission particles in Euro IV engine, value 1 is given to the smallest responses and others are fold-values. Each left column presents the relative responses per same mass dose (150µg/ml). Instead in each right column the response is first weighed with emission factor (mg/kWh) and then the relative response is calculated. 


	MIP-2	TNFá	Cytotoxicity	Apoptosis	Genotoxicity	Oxidative stress	
EN590	1.8	3.5	1.4	7.2	1.2	4.1	4.6	8.7	2.7	10.1	2.5	12.4	
EN590cat	1.5	1.2	1.6	3.1	1.0	1.4	2.2	1.6	1.5	2.2	1.8	3.5	
30% HVO	2.5	5.4	1.6	8.8	1.0	3.8	4.1	8.3	1.9	7.6	1.7	9.1	
100% HVO	2.1	2.4	1.4	4.1	1.0	2.1	3.0	3.3	1.1	2.4	1.3	3.8	
HVOcat	2.5	1.0	1.0	1.0	1.4	1.0	2.6	1.0	1.3	1.0	1.0	1.0	
30% RME	1.0	3.1	1.2	9.5	1.1	6.2	2.9	8.6	1.4	8.5	1.7	13.2	
100% RME	1.1	4.4	1.1	11.4	1.1	8.3	1.0	4.0	1.0	7.8	1.4	14.7	
